# Genome composition analysis of multipartite BNYVV reveals the occurrence of genetic re-assortment in the isolates of Asia Minor and Thrace

**DOI:** 10.1038/s41598-020-61091-2

**Published:** 2020-03-05

**Authors:** Canan Yüksel Özmen, Saber Delpasand Khabbazi, Afsaneh Delpasand Khabbazi, Songül Gürel, Rıza Kaya, Muhammet Çağrı Oğuz, Ferzat Turan, Fereshteh Rezaei, Umut Kibar, Ekrem Gürel, Ali Ergül

**Affiliations:** 1 Ankara University, Biotechnology Institute, 06135 Ankara, Turkey; 2 University of Tabriz, Department of Plant Protection, 51666 Tabriz, Iran; 3 Bolu Abant İzzet Baysal University, Department of Biology, 14030 Bolu, Turkey; 4 Sugar Institute, Department of Phytopathology, Etimesgut, 06930 Ankara Turkey; 5 Republic of Turkey Ministry of Agriculture and Forestry, Agriculture and Rural Development Support Institution, 06550 Ankara, Turkey; 6 Present Address: Başkent University, Institute of Transplantation and Gene Sciences, 06980 Kahramankazan, Ankara Turkey

**Keywords:** Biotechnology, Plant sciences

## Abstract

*Beet necrotic yellow vein virus* (BNYVV) is the cause of rhizomania, an important disease of sugar beet around the world. The multipartite genome of the BNYVV contains four or five single-stranded RNA that has been used to characterize the virus. Understanding genome composition of the virus not only determines the degree of pathogenicity but also is required to development of resistant varieties of sugar beet. Resistance to rhizomania has been conferred to sugar beet varieties by conventional breeding methods or modern genome engineering tools. However, over time, viruses undergo genetic alterations and develop new variants to break crop resistance. Here, we report the occurrence of genetic reassortment and emergence of new variants of BNYVV among the isolates of Thrace and Asia Minor (modern-day Turkey). Our findings indicate that the isolates harbor European A-type RNA-2 and RNA-3, nevertheless, RNA-5 is closely related to East Asian J-type. Furthermore, RNA-1 and RNA-4 are either derived from A, B, and P-types or a mixture of them. The RNA-5 factor which enhance the pathogenicity, is rarely found in the isolates studied (20%). The creation of new variants of the virus emphasizes the necessity to develop new generation of resistant crops. We anticipate that these findings will be useful for future genetic characterization and evolutionary studies of BNYVV, as well as for developing sustainable strategies for the control of this destructive disease.

## Introduction

Rhizomania is one of the most destructive soil-borne diseases of sugar beet (*Beta vulgaris* L.) worldwide. Since the first report of rhizomania^1^ numerous studies have reported the worldwide infection of sugar beet fields with this disease. Tamada and Baba^2^ first identified *Beet necrotic yellow vein virus* (BNYVV) as the cause of rhizomania when they isolated the virus from infected plants of sugar beet fields in Japan. This disease reduces sugar content by 8%, root yield up to 90%, and sugar yield up to 80%^3,4^. The BNYVV genome is multipartite and composed of four single-stranded RNA species designated as RNA-1, RNA-2, RNA-3, and RNA-4, coating with a 21-kDa protein^5^. In addition, a fifth RNA species (RNA-5) has been identified in some of the European and Asian BNYVV isolates^6–12^. RNA-1 and RNA-2, which contain 6746 and 4612 nt-long RNA species, respectively, encode viral “housekeeping” genes involved in virus replication, assembly, cell-to-cell movement and suppression of post transcriptional gene silencing^13,14^. Therefore, when the virus vector *Polymyxa betae* Keskin^15^ is not present, RNA-1 and RNA-2 are required for the maintenance of BNYVV in the environment^8,14,16^. RNA-3 consisting of a 1775 nt-long RNA species, is involved in viral pathogenicity^7,10,11,17,18^. RNA-4 (1431 nt) plays a key role in transmission of the virus by *P. betae*^7,11,13,19^. RNA-5 (1342–1347 nt in length) is associated with rhizomania severity, but is not required for virus survival^20,21^. Comparative studies revealed that the RNA-1, RNA-4, and RNA-5 contribute to the development of different rhizomania symptoms^7^. Moreover, the interaction between RNA-3, RNA-4 and RNA-5 increases rhizomania symptoms^11,20^. BNYVV is mainly divided into three pathogenic types designated as A, B and P types^4,8,12,17^. A and B are the most widespread types such that A-type is more prevalent than B-type virus and the P is a rare type throughout the world^3^. Type A is found in European countries as well as in the USA, China, and Japan, whereas, Type B occurs mainly in countries such as France, Germany, Sweden, Poland, China and Iran^6,10,22,23^. Type P is associated with RNA-5 and was first isolated from the city of Pithiviers, France^24^. However, RNA-5 species was previously described in East Asian isolates^20^. Studies also revealed that the J-type East Asian BNYVV isolates was different from French isolates for the length and the sequence of RNA-5^7,25^. With more than 340 thousand hectares of sugar beet harvesting area, Turkey is the world’s fifth largest sugar beet producing country (FAO stat, 2017) and rhizomania is known to cause serious economic losses in sugar beet production^26^.

In the current study, the BNYVV strains isolated from different provinces of Turkey with long history of sugar beet cultivation have been subjected to comprehensive genomic analyses of all the RNA components and further phylogenetic studies were carried out. According to our findings genetic reassortment between the European and East Asian A, B and P types has led to the emergence of new variants of BNYVV in the region of Thrace and Asia Minor (modern-day Turkey).

## Results

### DAS-ELISA and RT-PCR-based detections of BNYVV isolates

The double-antibody sandwich enzyme-linked immunosorbent assay (DAS-ELISA) results confirmed BNYVV infection of only 38 bait plants grown in collected soil samples (57.6% of all bait plants). The highest OD value was recorded 2.160 for samples of Kütahya; however, the other values of positive isolates ranged between 0.068–1.609 (Table 1). The DAS-ELISA assessments for all the plants grown in soil samples collected from Eskişehir, Kahramanmaraş, Kırşehir, Konya, Kütahya, Niğde, Sakarya, Tokat, Edirne and Elazığ provinces were positive. Among three provinces of Iğdır, Çorum and Bursa where the most number of sampling performed (7 samples from each), only 4, 3 and 2 soil samples were infected by BNYVV, respectively (Table 1).

**Table 1:** DAS-ELISA and RT-PCR based detection of BNYVV and RNA species in different sugar beet cultivation regions of Turkey.

No.	Code Soil sampling area		DAS-ELISA (Average Absorbance Value)	RT-PCR				
	Province (district)		(+/−)	RNA-1	RNA-2	RNA-3	RNA-4	RNA-5
1	TR-S1	Afyon (Şuhut)	−	−	−	−	−	−
2	TR-S2	Afyon (Çobanlar)	+ (0.217)	+	+	+	+	−
3	TR-S3	Afyon (Çay)	+ (0.186)	+	+	+	+	−
4	TR-S12	Aksaray (Center)	−	−	−	−	−	−
5	TR-S13	Aksaray (Yeşilova)	−	−	−	−	−	−
6	TR-S14	Aksaray (Yeşilova)	−	−	−	−	−	−
7	TR-S15	Aksaray (Yeşilova)	+ (0.214)	+	+	+	+	−
8	TR-S16	Amasya (Aydınca)	−	−	−	−	−	−
9	TR-S17	Amasya (Suluova)	−	−	−	−	−	−
10	TR-S19	Amasya (Suluova)	+ (0.689)	+	+	+	+	−
11	TR-S20	Amasya (Center)	+ (1.518)	−	−	−	−	−
12	TR-S21	Ankara(Ayaş)	−	−	−	−	−	−
13	TR-S22	Ankara (Ayaş)	+ (0.577)	+	+	+	+	−
14	TR-S23	Ankara (Ayaş)	+ (0.598)	+	+	+	+	−
15	TR-S24	Ankara (Polatlı)	+ (0.755)	+	+	+	+	−
16	TR-S28	Ankara (Temelli)	+ (0.410)	+	+	+	+	−
17	TR-S31	Burdur (Gölhisar)	−	−	−	−	−	−
18	TR-S32	Burdur (Gölhisar)	−	−	−	−	−	−
19	TR-S33	Burdur (Gölhisar)	+ (0.279)	+	+	+	+	−
20	TR-S37	Bursa (Yenişehir)	+ (1.384)	−	−	−	−	−
21	TR-S39	Bursa (Yenişehir)	+ (0.594)	−	−	−	−	−
22	TR-S40	Bursa (Karacabey)	−	−	−	−	−	−
23	TR-S42	Bursa (Karacabey)	−	−	−	−	−	−
24	TR-S43	Bursa(Mustafa Kemal)	−	−	−	−	−	−
25	TR-S44	Bursa (Mustafa Kemal)	−	−	−	−	−	−
26	TR-S45	Bursa (Mustafa Kemal)	−	−	−	−	−	−
27	TR-S46	Çorum (Osmancık)	+ (0.230)	−	−	−	−	−
28	TR-S47	Çorum (Osmancık)	−	−	−	−	−	−
29	TR-S48	Çorum (Osmancık)	+ (1.072)	+	+	+	+	−
30	TR-S49	Çorum (Osmancık)	+ (0.184)	+	+	+	+	+
31	TR-S50	Çorum (İskilip)	−	−	−	−	−	−
32	TR-S51	Çorum (İskilip)	−	−	−	−	−	−
33	TR-S57	Çorum (Sungurlu)	−	−	−	−	−	−
34	TR-S58	Denizli (Çivril)	+ (0.316)	+	+	+	+	−
35	TR-S59	Denizli (Çivril)	−	−	−	−	−	−
36	TR-S61	Edirne (Edirne)	+ (0.662)	+	+	+	+	−
37	TR-S67	Elazığ (Kovancılar)	+ (0.177)	+	+	+	+	−
38	TR-S76	Erzincan (Erzincan)	+ (0.844)	+	+	+	+	−
39	TR-S78	Erzincan (Erzincan)	−	−	−	−	−	−
40	TR-S79	Eskişehir (Sivrihisar)	+ (1.514)	+	+	+	+	+
41	TR-S82	Eskişehir (Research Station)	+ (1.401)	+	+	+	+	−
42	TR-S4	Iğdır	−	−	−	−	−	−
43	TR-S5	Iğdır	+ (0.839)	+	+	+	+	+
44	TR-S7	Iğdır	−	−	−	−	−	−
45	TR-S8	Iğdır	+ (0.387)	+	+	+	+	+
46	TR-S9	Iğdır	−	−	−	−	−	−
47	TR-S10	Iğdır	+ (0.640)	+	+	+	+	+
48	TR-S11	Iğdır	+ (0.189)	+	+	+	+	+
49	TR-S86	Kahramanmaraş (Center)	+ (1.609)	+	+	+	+	−
50	TR-S91	Kastamonu (Taşköprü)	+ (0.159)	+	+	+	+	−
51	TR-S93	Kastamonu(Center)	−	−	−	−	−	−
52	TR-S105	Kırklareli (Uzunköprü)	+ (0.068)	+	+	+	+	−
53	TR-S107	Kırklareli (Alpullu)	−	−	−	−	−	−
54	TR-S113	Kırşehir (Dedeli)	+ (0.986)	+	+	+	+	−
55	TR-S117	Konya (Çumra)	+ (0.431)	+	+	+	+	−
56	TR-S119	Konya (Karapınar)	+ (1.017)	+	+	+	+	+
57	TR-S121	Konya (Ilgın)	+ (0.159)	+	+	+	+	−
58	TR-S125	Kütahya (Simav)	+ (2.160)	+	+	+	+	−
59	TR-S129	Niğde (Center)	+ (1.106)	+	+	+	+	−
60	TR-S137	Sakarya (Budaklar)	+ (0.079)	+	+	+	+	−
61	TR-S139	Samsun (Çarşamba)	−	−	−	−	−	−
62	TR-S141	Tokat (Turhal)	+ (1.240)	+	+	+	+	−
63	TR-S146	Yozgat (Sarıkaya)	−	−	−	−	−	−
64	TR-S158	Yozgat (Boğazlıyan)	−	−	−	−	−	−
65	TR-S156	Yozgat (Çekerek)	+ (0.285)	+	+	+	+	−
66	TR-S160	Yozgat (Akdağmadeni)	+ (0.896)	+	+	+	+	−
67	−	Positive Control 1	+ (2.196)	+	+	+	+	+
68	−	Positive Control 2	+ (2.208)	+	+	+	+	+
69	−	Negative Control 1	− (0.012)	−	−	−	−	+
70	−	Negative Control 2	− (0.019)	−	−	−	−	−

+/−: depicts the presence/absence of BNYVV infection and RNA species in bait plants grown in soil samples

**Figure 1:**
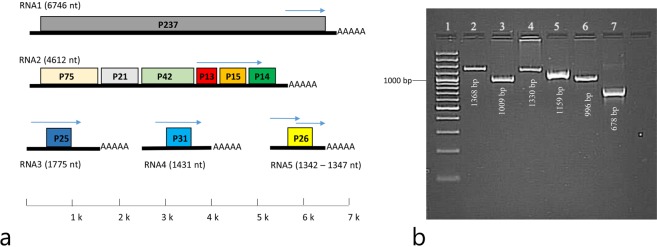
(**a**) Genome structure of BNYVV comprising five different RNA species. Each of the RNA-1–5 components contain a single or multiple ORFs. The arrows indicate the region amplified by primers. (**b**) Amplification of RNA-1–5 was optimized using primers designed. Lane 1 GeneRuler100bp Plus DNA Ladder (Thermo Scientific™), lanes 2 – 5 represent RNA-1–4, Lanes 6–7 represent two different amplifications of RNA-5.

Reverse transcription-polymerase chain reaction (RT-PCR) assay was successfully optimized to amplify the desired fragments using positive control isolates (Fig. 1b). Based on RT-PCR, BNYVV isolates were detected in only 34 of the bait plants (51% of all bait plants). The majority of the BNYVV isolates (27 isolates) contained RNA-1-4, while only 7 isolates (20%) contained all five RNA species, indicating the rarity of RNA-5 in Turkish BNYVV isolates (Table 1).

### Genome analysis and typology of the BNYVV isolates

#### RNA-1

**Figure 2:**
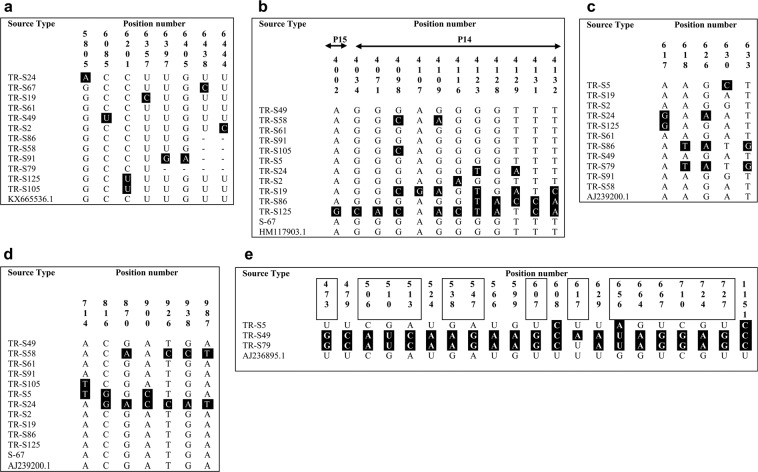
Nucleotide sequence alignment of RNA-1–5 of the isolates. Analyses revealed unique polymorphism occurred at: (**a**) eight positions in RNA-1(P237) between 5427–6520 nt; (**b**) twelve positions in RNA-2 (within the ORFs P15 and P14) between 3700–4140 nt; (**c**) five positions in RNA-3 (P25) between 420–1000 nt; (**d**) seven positions in RNA-4 (P31) between 381–1280 nt; (**e**) twenty-one positions in the complete coding sequence of RNA-5 (P26). Nucleotide sequences of RNA-1–5 were adjusted according to KX665536.1, HM117903.1, AJ239200.1, AJ239200.1 and AJ236895.1, respectively. Nucleotides highlighted in black boxes are novel occurrence of polymorphism. Dashes signify missing nucleotides which were not analyzed. Nucleotide variations of RNA-5 that cause amino acid replacements are boxed-in respective nucleotide positions.

**Figure 3:**
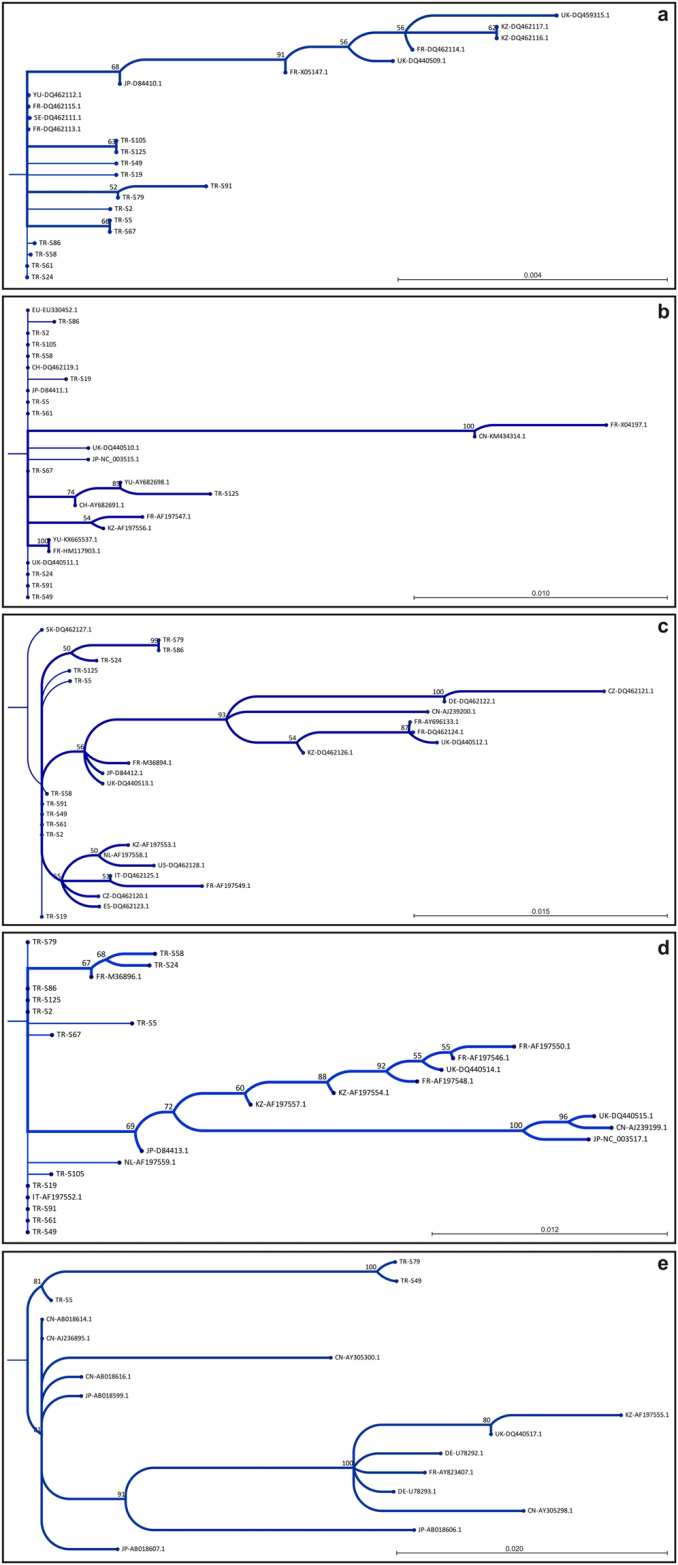
Phylogenetic relationship of genomic compartments (RNA-1–5) of BNYVV and sequences available in Genbank. Trees were created using Neighbor-Joining method and Jukes-Cantor distance algorithm. Numbers at the branch junctions represent the percent of trees out of 100 replications. (**a**) RNA-1, (**b**) RNA-2, (**c**) RNA-3, (**d**) RNA-4 and (**e**) RNA-5. Accession numbers and detailed information about the reference sequences are given in Sup. 1.

Isolates representing different regions (TR-S24, TR-S67, TR-S19, TR-S61, TR-S49, TR-S5, TR-S2, TR-S86, TR-S58, TR-S91, TR-S79, TR-S125, TR-S105) were selected to study the nucleotide sequences of RNA-1 (Table 1). Analysis of RNA-1 (P237) in the coding region of 5427–6520 and comparing the results with available sequences in the NCBI database revealed the incident of novel nucleotide polymorphism among the isolates in 8 positions (Fig. 2a). Similarity assessments indicated that most of the isolates are highly similar to the A-type isolates of S, F-Pi76 and Yu2 earlier reported from Japan (D84410.1), France (DQ462115) and Yugoslavia (DQ462112), respectively. Isolates such as TR-S24, TR-S86, TR-S58, TR-S91, TR-S125, TR-S105 and TR-S79 are highly similar to the A-type isolates S, F-Pi76 and Yu2 (99.44–100%). However, in other isolates (TR-S67, TR-S61, TR-S49, TR-S5, TR-S2) a complex structure was revealed as these resemble both the P and A-type isolates of S, F-Pi76 and Yu2 (99.35–99.66%) (Table 2). Phylogenetic studies indicated that the new isolates are in close relationship with Japanese Isolate S (bootstrap value 68%), and isolates S8, Yu2, F-Pi72 and F-Pi76 from Sweden, Yugoslavia and France with the support of bootstrap lower than 50% (Fig. 3a; Sup. 1).

**Table 2:** Typology of Turkish BNYVV isolates based on similarity analysis of RT-PCR sequences.

RNA species	Analyzed sequence (bp)	Soil sample code	Similarity analysis of RT-PCR sequence (%)	Type	Accession no.	Isolate	Country
RNA-1	5427–6520	TR-S24	99.72	A	D84410.1	S F-Pi76 Yu2	Japan France Yugoslavia
		TR-S67	99.55	A/P	D84410.1/DQ462115/DQ462112		
		TR-S19	99.54	A	D84410.1		
		TR-S61	99.66	A/P	DQ462115/DQ462112		
		TR-S49	99.55	A/P	D84410.1/DQ462115/DQ462112		
		TR-S5	99.35	A/P	D84410.1/DQ462115/DQ462112		
		TR-S2	99.55	A/P	D84410.1/DQ462115/DQ462112		
		TR-S86	99.89	A	D84410.1		
		TR-S58	99.89	A	D84410.1		
		TR-S91	99.69	A	D84410.1		
		TR-S79	100	A	D84410.1		
		TR-S125	99.44	A	D84410.1		
		TR-S105	99.63	A	D84410.1		
RNA-2	3700–4140	TR-S24	100	A	AY682691.1 AY682698.1	Rio Zurich A14	Switzerland Yugoslavia
		TR-S67	100				
		TR-S19	100				
		TR-S61	100				
		TR-S49	100				
		TR-S5	100				
		TR-S2	100				
		TR-S86	100				
		TR-S58	100				
		TR-S91	100				
		TR-S24	100				
		TR-S125	100				
		TR-S105	100				
RNA-3	420–1000	TR-S24	99.45	A	DQ462127.1	Sl7	Slovakia
		TR-S19	99.83				
		TR-S61	99.83				
		TR-S49	99.83				
		TR-S5	99.66				
		TR-S2	99.83				
		TR-S86	99.16				
		TR-S58	99.79				
		TR-S91	99.83				
		TR-S79	99.16				
		TR-S125	99.66				
RNA-4	381–1280	TR-S24	97.31	A/B	AF197552.1/M36896.1	I-12 F2	Italy France
		TR-S67	99.88	A	AF197552.1		
		TR-S19	99.88	B	M36896.1		
		TR-S61	100	B	M36896.1		
		TR-S49	100	B	M36896.1		
		TR-S5	99.45	A	AF197552.1		
		TR-S2	99.76	B	M36896.1		
		TR-S86	100	A/B	AF197552.1/M36896.1		
		TR-S58	99.37	B	M36896.1		
		TR-S91	100	A/B	AF197552.1/M36896.1		
		TR-S79	99.88	B	M36896.1		
		TR-S125	100	A/B	AF197552.1/M36896.1		
		TR-S105	99.88	B	M36896.1		
RNA-5	420–1160 (ORF)	TR-S5	99.58	I-A (J-type)	AB018614.1	CH2	China
		TR-S49	97.11				
		TR-S79	97.11

#### RNA-2

Isolates of TR-S49, TR-S58, TR-S61, TR-S91, TR-S105, TR-S5, TR-S24, TR-S2, TR-S19, TR-S86, TR-S125, TR-S67 were studied for P13, P14 and P15 open reading frames (ORF) corresponding to transport and suppressor associated proteins in the positions of between 3700–4140 nt. Although no novel nucleotide variation was identified in P13, ORF sequences of P14 and P15 contained several nucleotide variations that were not previously reported (Fig. 2b). RNA-2 sequence analyses designated the isolates as A-type isolates (Table 2). According to the matrix analyses of RNA-2, isolates were identical to the Rio Zurich and A14 isolates reported from Switzerland (AY682691.1) and Yugoslavia (AY682698.1). Phylogenetic analyses displayed that TR-S67 and TR-S125 are related to the isolates A142 (AY682698.1) and Rio Zurich (AY682691.1) with the support of bootstrap value over 70%. In addition, the remaining isolates are closely related to the isolates of Japan (S), Switzerland (Ch23) and UK (MH) with the bootstrap value lower than 50% (Fig. 3b; Sup. 1).

#### RNA-3

Studying the isolates of TR-S5, TR-S19, TR-S2, TR-S24, TR-S125, TR-S61, TR-S86, TR-S49, TR-S79, TR-S91, TR-S58 for RNA-3 (P25) revealed the occurrence of unique nucleotide polymorphisms between the nucleotide positions of 420–1000 (Fig. 2c). Based on matrix analyses similarity ratios calculated and accordingly isolates highly (99.16–99.83%) resemble Sl7 isolate reported from Slovakia (DQ462127.1) (Table 2). Results designated RNA-3 as A-type and furthermore, a close phylogenetic relationship was identified between current isolates and isolates of Slovakia (bootstrap < 50%), UK and Spain (bootstrap > 50%) (Fig. 3c).

#### RNA-4

RNA-4 (P31) analyses of the selected isolates (TR-S49, TR-S58, TR-S61, TR-S91, TR-S105, TR-S5, TR-S24, TR-S2, TR-S19, TR-S86, TR-S125 and TR-S67) unveiled the incidence of unique polymorphism in the nucleotide position of 381–1280 (Fig. 2d). According to matrix analyses of RNA-4 sequences, the isolates were either highly resembling or identical to the Italian isolate I-12 (AF197552.1) and French isolate F2 (M36896.1). Interestingly, some of the isolates (TR-S86, TR-S91, and TR-S125) were identical to both of the I-12 and F2 isolates (Table 2). According to RNA-4-based similarity assessments, TR-S19, TR-S61, TR-S49, TR-S2, TR-S58, TR-S79 and TR-S105 isolates were designated as B-type, however, TR-S67 and TR-S5 isolates were A-type (Table 2). According to phylogenetic studies, some of the isolates were in a close relationship with the French isolate F2 and some others were related to the Italian isolate I-12. This finding was consistent with the results of the matrix analysis of nucleotide sequence similarities (Fig. 3d).

#### RNA-5

Sequence analysis of RNA-5 (P26) in the coding region (420–1160 nt) showed that TR-S49 and TR-S79 isolates were notably different from previous ones, unlike TR-S5. Despite the presence of polymorphism in TR-S5, the nucleotide sequence is the closest (99.58%) to the Chinese isolate CH2 (AB018614.1). Nevertheless, the genetic distance between TR-S49 and TR-S79 isolates and previously reported sequences was higher (97.11%). The results showed that the studied isolates harbored P26 similar to East Asian J-type (Table 2), but there were a significant number of nucleotide variations. More than 66% of these nucleotide variations lead to amino acid replacements, emphasizing the genetic difference of TR-S49 and TR-S79 (Fig. 2e). Phylogenetic analyses revealed a close genetic relationship between these isolates and those previously reported. Therefore, all isolates were placed in one cluster and the highest relationship was noted with East Asian isolates with bootstrap value over 80% (Fig. 3e; Sup. 1).

## Discussion

To control losses incurred by rhizomania, cultivation of resistant sugar beet cultivars developed through conventional breeding methods and transgenic techniques is considered an effective approach. Emergence and evolution of novel variants of BNYVV threaten sustainable crop production and yield in resistant crops, however, these variants are not widespread yet^3^. ELISA, RT-PCR and restriction fragment length polymorphisms (RFLP) analyses have been widely used in detection and studies of BNYVV structure. ELISA is a routine serological assay used in diagnosis of BNYVV in plants, although concentration of the virus, soil temperature and the type of the sampling tissue affect the efficiency of this method^27,28^. Therefore, the ELISA test is less efficient particularly when virus concentration is low in infected plants^22,29^.

The accuracy of the ELISA is less than molecular-based detection methods, however, being fast, cheap and the presence of commercial kits make the ELISA a preferred assay for the routine detection of BNYVV. RT-PCR is a more accurate tool compared to ELISA and is considered a more reliable method in virus detection studies^22,30–34^ hence, it is increasingly used in the detection of BNYVV^35–37^. The use of nucleic acid-based methods such as RT-PCR and qRT-PCR has increased the sensitivity of the virus detection by up to 100–10,000 times^27^.

Absorbance values of ELISA are closely related to rhizomania disease index score, so the higher the disease index scores, the greater the absorbance values. The ELISA result is considered positive only if the absorbance values of the samples are more than three times the value in the negative control samples^38^. In this study, the ELISA values recorded for negative controls were below 0.02 (0.012–0.019) therefore values over 0.06 were accepted as positive samples (Table 1). The ELISA results of bait plants indicate the infection of BNYVV in 57.6% of the sampling regions. A wide range of absorbance values among the positive samples of different regions (from 0.068 to 2.160) could be due to the different severity index of the disease in these areas. RT-PCR analyses of bait plants verified 89% of the ELISA results which could be relevant to the higher accuracy of the RT-PCR. According to RT-PCR results, 51% of the samples are positive for BNYVV and the majority of the positive samples lack the RNA-5 species thus indicating the rare distribution of the higher virulent BNYVV pathotypes in the sampling regions (Table 1). The results of this study corroborate previous studies reporting the prevalence of BNYVV in sugar beet fields of Turkey^39^.

Since the first record of BNYVV infection in Turkey^40^ several research works have diagnosed the disease in sugar beet fields of the country; however, only a few studies have partially characterized the virus^39,41–43^. Partial sequence analyses of RNA-3 isolated from Tokat province in the mid Black Sea region of Anatolia described some differences in the amino acid sequence of the Turkish isolates^43^. Previous nucleotide analyses of RNA-3 and RNA-5 of Turkish isolates assigned these species as A-type and East Asian J-type^39,43^ respectively. RFLP-based analyses of the isolates from the northern and central parts of Turkey designated RNA-2 and RNA-3 as A-type species^42,43^ nevertheless, RFLP results of some isolates did not match the expected band profile which was the inspiration for the current study to examine the presence of polymorphism among Turkish isolates. RNA-1–5 analyses of BNYVV isolated from different regions of Thrace and Anatolia (modern-day Turkey) revealed a unique polymorphism which could be the probable causes of the failure of RFLP analysis in the previous study^42^. Our results are in accordance with previous studies that assigned RNA-2–3 as A-type and RNA-5 as East Asian J-type. Furthermore, this study found that RNA-1 was derived either only from A type BNYVV or from mixture of P and A-types as some of the isolates were harboring RNA-1 highly resembling both European and Japanese P and A-types (Table 2). However, the majority of RNA-1 species were closely related to the Japanese A-type strains. Based on RNA-4 analysis, isolates were mostly designated as either European A or B-types. However, there were some isolates very similar or identical to both A and B-types that seems to be derived from mixture of A and B-types. The finding that multipartite genome of BNYVV could be comprised of RNA species with different sources has rarely been reported^12,44,45^. Studying the isolates from the areas located in a borderline region located between the distribution areas of different BNYVV types indicated the incident of genome re-assortments^45^. Although mixed infection of different virus types is rarely observed^46^ it might provide the required condition for this genetic alteration. Genome re-assortments lead to the emergence of new variants of the virus that may overcome the resistance of cultivars.

Among different pathogenic types of BNYVV, strains containing RNA-5 are more destructive than those lacking the fifth RNA species^20,21^. The worldwide distribution of RNA-5-containing isolates is less common than those lacking it. Studying the isolates of 24 provinces in Turkey revealed the rare occurrence of BNYVV strains harboring RNA-5 in infected fields which is in agreement with the earlier report^42^. RNA-5 was detected only in four provinces of Iğdır, Eskişehir, Çorum and Konya and based on RT-PCR only 7 out of 34 soil samples infected with BNYVV were harboring RNA-5 species (20%). Moreover, Iğdır province in Eastern Anatolia had the highest RNA-5 occurrence (57%) among the studied regions. Analysis of RNA-5 in the coding sequences indicated that although all the TR-S5, TR-S49 and TR-S79 isolates were closely related to East Asian J-type, there was a notable nucleotide and amino acid difference between them (Table 2).

In conclusion, this study aimed to carry out sequence analyses of the BNYVV genomic compartments and typology of RNA-1–5 based on pairwise identity and similarity assessments along with phylogenetic relationships. We detected the B type RNA species in the Anatolian region where BNYVV isolates were designated as A type virus in previous studies. Furthermore, our study reveals the occurrence of genomic re-assortments between P, B and A-type viruses which is the first report. The emergence of new variants of the virus threatens the sustainable production of sugar beet in the world, therefore, these findings will contribute to the sustainable control of BNYVV.

## Methods

### Collection of soil samples and virus inoculations

**Figure 4:**
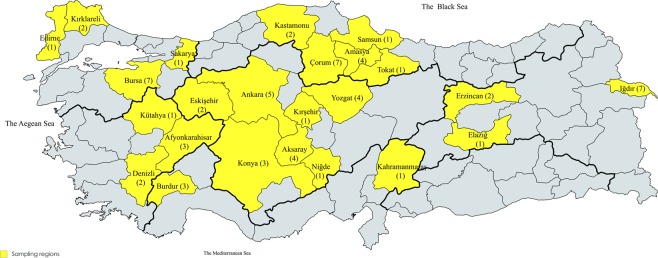
Distributions of BNYVV sampling regions within Turkey (highlighted with yellow). A total of 66 soil samples were collected from 24 provinces. The number of soil samples collected from each province were given in brackets (The map is created with the online mapchart.net application version 7.9 available at https://mapchart.net/turkey.html).

Soil samples were collected from 66 sugar beet cultivation regions in 24 provinces of Turkey and dried out at room temperature (Fig. 4). Two different soil samples known to be infested with BNYVV were supplied by the Sugar Research Institute (Ankara, Turkey) were included in the study as positive controls for RNA-1–5 components. The samples were pulverized and sieved through 2 mm-pores, mixed with sand (3:1 ratio of soil and sand) and filled into plastic pots (1.5 kg per each pot). The experiment was performed in 3 replicates. *Beta vulgaris* L. cv. Ansa was selected as the BNYVV-susceptible cultivar to be deployed in virus infection experiments of bait plants. In each pot, 30 seeds of bait plants were planted and kept at 25 °C and 16/8-hour light/dark period for 10 weeks. Following germination, 15 plantlets were randomly collected from each pot. The roots were detached, washed with distilled water and dried out. The root pieces collected from each soil sample were compiled and ground with porcelain mortar and pestle using liquid nitrogen. After grinding, one gram of crushed root tissue was used in the ELISA test and the remaining amount was stored at −80 °C to be utilized for RNA isolations and RT-PCR assays.

### Enzyme-linked immunosorbent assays

The DAS-ELISA was performed to quantify the BNYVV accumulation in the samples using the Bioreba AG Kit (Catalog No. # 160175). The DAS-ELISA assay was carried out according to the manufacturer’s recommendations. Bio-Rad Novapath Microplate Reader was used to measure absorbance values of the samples at 405 nm wavelength. The data were analyzed following the Meunier^47^ procedure. The DAS-ELISA test was carried out separately for each replicate and average values were calculated (Table 1).

### RNA isolations

Total RNA was isolated from root tissues using the TRI Reagent™ kit using manufacturer’s recommendations (Bioshop Inc., Canada). RNA concentrations were analyzed by a NanoDrop-1000 spectrophotometer (NanoDrop Technologies, Llc., Wilmington, USA) and the quality was checked by 1% agarose gel electrophoresis and visualizing under UV (GeneGenius Gel Imaging system).

### Primer designs and RT-PCR assays

BNYVV nucleotide sequences were retrieved from the GeneBank and highly conserved regions were used to design PCR primers (Fig. 1a). The primer sets specific for BNYVV genomic components (RNA-1–5) were designed using the NCBI Primer Designing tool (Table 3).

**Table 3:** Primer sequences used for amplifications of different RNA components of BNYVV.

BNYVV components	Primer Sequence	Product length (bp)	Annealing temperature (°C)
RNA-1	F: 5′ TGCATGACATGGTCGCAAAA 3′ R: 5′ TGGTACAATTCACACCCAGTCA 3′	1368	60
RNA-2	F: 5′ ACTAGAGCTCGTAAGGGTGGT 3′ R: 5′ ACCGCGATGGTGAACAATTTC 3′	1009	60
RNA-3	F: 5′ ACCGACCAAATCCAAGCGAG 3′ R: 5′ CGCTACTGCACACTCTTTACCA 3′	1330	55
RNA-4	F: 5′ ACCTTAGATTCACGAGCCGC 3′ R: 5′ TGGTACATTTCACACCCAGTCA 3′	1159	60
RNA-5	F: 5′ AGTACCGCTGTTCTAAGTGACG 3′ R: 5′ TCGGACTGCAACATAAAAGCAC 3′ ------------------------------------------------------- F: 5′ TGTTGCCACAAATTTTCCAGGT 3′ R:5′ GTCAATACACTGACAGAGAACCCT 3′		55

The NG dART RT kit (Eurex, Poland) was used to perform the first strand cDNA synthesis and RT-PCR assay. Prior to screening, positive controls were utilized to optimize the amplification of the RNA species. cDNA concentrations and quality were assessed by a NanoDrop-1000 Spectrophotometer. PCR amplifications were performed in a 25 µL reaction mixture containing 200 ng of cDNA, 10 pmol of each primer, 2.5 mM dNTPs, 0.5 units GoTaq DNA polymerase (Promega, Madison, WI, USA) and 1.5 mM MgCl_2_. PCR was initiated with a denaturing step of 3 min at 94 °C followed by 35 cycles including 94 °C for 1 min (denaturation), 60 °C for 2 min (annealing) and 72 °C for 2 min (extension), and the final extension period of 10 min at 72 °C. PCR products were electrophoresed on 1.5% agarose gel and the GeneGenius Gel Imaging system was used to visualize the amplicons.

### Sequence analyses

Some of the isolates were selected based on ELISA and RT-PCR results to undergo further sequence and phylogenetic analyses. The resulting PCR products of RNA-1–5 species were purified from agarose gels after electrophoresis and double sequenced by the Sanger dideoxy method (BM Labosis Ltd. Co., Ankara, Turkey). Sequence analyses were performed for RNA 1–5 species in comparison with relevant sequences retrieved from the GeneBank. The sequences obtained were initially subjected to the NCBI (National Center for Biotechnology Information) Blast analysis. Sequence comparisons were conducted using the CLC Sequence Viewer 7.6.1 workbench (CLC Bio-Qiagen, Aarhus, Denmark). Phylogenetic trees were constructed to study the evolutionary relationships. CLC Sequence Viewer 7.6.1 program was used to perform the phylogenetic analysis^48^. Trees were created by the Neighbor Joining method using the Jukes-Cantor distance algorithm and bootstrap support of 100. Pairwise identity and similarity of nucleotide sequences were calculated through matrix analyses performed by a SIAS online tool (Immunomedicine Group, UCM, Spain).

## Supplementary information

Supplementary information.
