# Supplementary material for: Genome composition analysis of multipartite BNYVV reveals the occurrence of genetic re-assortment in the isolates of Asia Minor and Thrace
Source: Sci Rep. 2020 Mar 5;10:4129. doi: 10.1038/s41598-020-61091-2 (PMC7058063; doi:10.1038/s41598-020-61091-2)
Supplement: Supplementary file 1 — Supplementary information. [file 41598_2020_61091_MOESM1_ESM.docx]

**Supplementary 1.** List of RNA species used in this study with related accession numbers and geographical origions

| **RNA species** | **Accession numbers** | **Codes given to isolates in this study** | **Isolate/Strain** | **Country** |
| --- | --- | --- | --- | --- |
| **RNA-1** | DQ462112.1 | YU-DQ462112.1 | Yu2 | Yugoslavia |
|  | DQ462115.1 | FR-DQ462115.1 | F-Pi76 | France |
|  | DQ462113.1 | FR-DQ462113.1 | F-Pi72 | France |
|  | DQ462117.1 | KZ-DQ462117.1 | Kas3 | Kazakhstan |
|  | DQ462116.1 | KZ-DQ462116.1 | Kas2 | Kazakhstan |
|  | DQ459315.1 | UK-DQ459315.1 | MH | United Kingdom |
|  | DQ440509.1 | UK-DQ440509.1 | FF | United Kingdom |
|  | DQ462114.1 | FR-DQ462114.1 | F-Pi75 | France |
|  | X05147.1 | FR-X05147.1 | F2, F13 | France |
|  | D84410.1 | JP-D84410.1 | S | Japan |
|  | DQ462111.1 | SE-DQ462111.1 | S8 | Sweden |
| **RNA-2** | DQ440510.1 | UK-DQ440510.1 | FF | United Kingdom |
|  | NC_003515.1 | JP-NC_003515.1 | S | Japan |
|  | X04197.1 | FR-X04197.1 | F13 | France |
|  | KM434314.1 | CN-KM434314.1 | Hu3 | China |
|  | AY682698.1 | YU-AY682698.1 | A14 2 | Yugoslavia |
|  | AY682691.1 | CH-AY682691.1 | Rio Zurich | Switzerland |
|  | DQ440511.1 | UK-DQ440511.1 | MH | United Kingdom |
|  | AF197547.1 | FR-AF197547.1 | F75 | France |
|  | AF197556.1 | KZ-AF197556.1 | Kas3 | Kazakhstan |
|  | DQ462119.1 | CH-DQ462119.1 | Ch23 | Switzerland |
|  | KX665537.1 | YU-KX665537.1 | PV0467/Yu2 | Yugoslavia |
|  | EU330452.1 | EU-EU330452.1 | S8 | Europe |
|  | HM117903.1 | FR-HM117903.1 | Pithivier | France |
|  | D84411.1 | JP-D84411.1 | S | Japan |
| **RNA-3** | DQ462127.1 | SK-DQ462127.1 | Sl7 | Slovakia |
|  | DQ462121.1 | CZ-DQ462121.1 | Cz4 | CzechRepublic |
|  | DQ462122.1 | DE-DQ462122.1 | D15 | Germany |
|  | AJ239200.1 | CN-AJ239200.1 | NM | China |
|  | AY696133.1 | FR-AY696133.1 | EP2 | France |
|  | DQ462124.1 | FR-DQ462124.1 | F-Pi88 | France |
|  | DQ440512.1 | UK- DQ440512.1 | MH | United Kingdom |
|  | DQ462126.1 | KZ-DQ462126.1 | Kas3 | Kazakhstan |
|  | M36894.1 | FR-M36894.1 | - | France |
|  | D84412.1 | JP-D84412.1 | S | Japan |
|  | DQ440513.1 | UK-DQ440513.1 | FF | United Kingdom |
|  | DQ440512.1 | UK-DQ440512.1 | MH | United Kingdom |
|  | AF197553.1 | KZ-AF197553.1 | Kas2 | Kazakhstan |
|  | AF197558.1 | NL-AF197558.1 | N7 | Netherlands |
|  | DQ462128.1 | US-DQ462128.1 | U7 | USA |
|  | DQ462125.1 | IT-DQ462125.1 | I-15 | Italy |
|  | AF197549.1 | FR-AF197549.1 | F76 | France |
|  | DQ462120.1 | CZ-DQ462120.1 | Cz3 | CzechRepublic |
|  | DQ462123.1 | ES-DQ462123.1 | E11 | Spain |
| **RNA-4** | M36896.1 | FR-M36896.1 | F2 | France |
|  | AF197550.1 | FR-AF197550.1 | F76 | France |
|  | AF197546.1 | FR-AF197546.1 | F72 | France |
|  | DQ440514.1 | UK-DQ440514.1 | MH | United Kingdom |
|  | AF197548.1 | FR-AF197548.1 | F75 | France |
|  | AF197554.1 | KZ-AF197554.1 | Kas2 | Kazakhstan |
|  | AF197557.1 | KZ-AF197557.1 | Kas3 | Kazakhstan |
|  | DQ440515.1 | UK-DQ440515.1 | FF | United Kingdom |
|  | AJ239199.1 | CN-AJ239199.1 | NM | China |
|  | NC_003517.1 | JP-NC_003517.1 | S | Japan |
|  | D84413.1 | JP-D84413.1 | S | Japan |
|  | AF197559.1 | NL-AF197559.1 | N7 | Netherlands |
|  | AF197552.1 | IT-AF197552.1 | I12 | Italy |
| **RNA-5** | AB018614.1 | CN-AB018614.1 | CH2 | China |
|  | AJ236895.1 | CN-AJ236895.1 | Huhhot | China |
|  | AB018616.1 | CN-AB018616.1 | CY1 | China |
|  | AY305300.1 | CN-AY305300.1 | Ningxia | China |
|  | AB018599.1 | JP-AB018599.1 | D104 | Japan |
|  | AF197555.1 | KZ-AF197555.1 | Kas2 | Kazakhstan |
|  | DQ440517.1 | UK-DQ440517.1 | MH | United Kingdom |
|  | U78292.1 | DE-U78292.1 | F28 | Germany |
|  | AY823407.1 | FR-AY823407.1 | P | France |
|  | U78293.1 | DE-U78293.1 | F72 | Germany |
|  | AY305298.1 | CN-AY305298.1 | Xinjiang | China |
|  | AB018606.1 | JP-AB018606.1 | S12 | Japan |
|  | AB018607.1 | JP-AB018607.1 | S13 | Japan |
